# Unintentional Drownings in Pediatric Populations: Injury Prevention in the Post-COVID-19 Era

**DOI:** 10.7759/cureus.38264

**Published:** 2023-04-28

**Authors:** Shamieh Banihani, Ian Waldrop, Manpreet K. Singh, Olivia Vukcevich, Nicholas W Sheets, David Plurad

**Affiliations:** 1 School of Medicine, University of California, Riverside, Riverside, USA; 2 Trauma and Acute Care Surgery, Riverside Community Hospital, Riverside, USA

**Keywords:** child injury prevention, pediatric preventative medicine, trauma and injury prevention, pediatric injuries, drowning

## Abstract

Unintentional injuries are one of the leading causes of death in Americans. A large proportion of these deaths are attributable to accidental drownings and falls, both of which oftentimes take place in or around swimming pools and swimming pool-related apparatuses such as diving boards. The American Academy of Family Physicians (AAFP) has reported drowning incidents as the most common injury-related cause of death in children ages one to four years. Although the AAFP has outlined steps to take to prevent drownings, there has not been a current large-scale study illustrating the effectiveness of these strategies with regard to their effect on the prevalence of swimming pool drowning cases in the last 10 years. Thus, we aim to utilize the National Electronic Injury Surveillance System (NEISS) database to uncover these rates, which can ultimately help aid in the reevaluation of current recommended guidelines.

## Introduction

Unintentional injuries are one of the leading causes of death in Americans. A large proportion of these deaths are attributable to accidental drownings and falls, both of which oftentimes take place in or around swimming pools and swimming pool related apparatuses such as diving boards. A European study published in 2009 reported 34 cervical spine injuries resulting from swimming pool diving accidents and found that 97% of these injuries were sustained by young men with a mean age of 27 years [1]. This study also highlighted the consequences of such injuries on the well-being of these patients beyond physical and onto socio-professional outcomes, including dissolutions of personal relationships and long term neurological complications that resulted in lost jobs [1]. 

Another study published in 2018 in the International Journal of Pediatric Otorhinolaryngology conducted a larger scale assessment of swimming pool diving related traumas, namely pediatric swimming and diving related facial traumas, using the National Electronic Injury Surveillance System (NEISS) database during the 10 year period between 2007 and 2016. They found that the most common facial fracture involved the nasal bone (87%) [2]. Despite this focus on diving related incidents, there have been very few studies that assess the prevalence of swimming pool traumas related to drowning. The American Academy of Family Physicians (AAFP) reported drowning as the most common injury-related cause of death in children ages one to four years (3). Although the AAFP has outlined steps to prevent drowning in the 2016 issue of American Family Physician, there has not been a current large scale study illustrating the prevalence of swimming pool drowning cases in the last 10 years. Thus, we aim to utilize the NEISS database to uncover these rates, which can ultimately help aid in the reevaluation of current recommended guidelines.

## Materials and methods

Data source

A retrospective study was conducted utilizing the National Electronic Injury Surveillance System (NEISS) to collect data regarding injuries involving drowning. The NEISS is a nationally representative sample of approximately 100 emergency departments located in the United States. In particular, its dataset represents a weighted probability sample of more than 5000 emergency departments in the United States and its territories, which allows users to collect data on consumer-product related injury rates and their characteristics. This includes demographic information such as age, gender, and ethnicity, information about the injury including diagnosis, location of injury, and disposition, and a brief narrative regarding the events surrounding the injury. Data is collected from on-site, trained coders from the electronic emergency department records, and this is transmitted back to quality assurance coders at the NEISS program, who go on to review and complete the coding before publishing in the NEISS. The database is available for public access and is provided by the Consumer Product Safety Commission (CPSC), which initially established this database to help governmental agencies monitor consumer-product related products.

Database query and collection

National estimates were not utilized in this study, and instead, were calculated based on patients with existing narratives in the NEISS system. This database provides secondary data and all patients recorded within the NEISS database who met the inclusion criteria were included in this study. The NEISS was queried for all drowning related injuries between the years 2012 and 2021. Patients were included if they met the age range requirements of 0-9 years of age. Data collected included general demographic information such as age, sex, and race. The mechanism of injury was collected based on the primary product codes linked to the injury including above ground swimming pools (code 3221), built in swimming pools (code 3251), diving or diving boards (code 1278), flotation toys (code 3279), portable swimming pool (code 5043), swimming (activity, apparel, or equipment {code 3274}), swimming pool equipment (code 3262), swimming pool slides (code 1277), and swimming pools not specified (code 1284). Diagnosis included was submersion (code 69). All body parts and their distinct codes were included. Patient disposition was coded as treated and released, treated and transferred, treated and admitted, held for observation, left either against medical advice (AMA) or without being seen, or fatality, which includes death prior to ED arrival, death in the ED, and death after admission. Location of injury was defined as locale where the injury took place and no limiting parameters were set within the database query. All unique information regarding locations of injury were collected.

Statistical analysis

General descriptive analyses were performed utilizing IBM SPSS Statistics version 27.0 (IBM Corp., Armonk, NY). All descriptive statistics were based on raw data from actual cases involving those treated in NEISS emergency departments. For comparing categorical variables such as groups comprised of sex differences, Chi-square analyses were performed with Microsoft Excel. P-values <0.05 were described as significant.

IRB exemption 

The NEISS is a publicly available database that utilizes anonymous patient information. This study qualifies as nonhuman subject research and as per Hospital Corporation of America (HCA) Healthcare's institutional review board (IRB) policy, is exempt from IRB review. 

## Results

A NEISS query returned 2122 results. Drowning injuries were more common in males (59.8%) compared to females (40.2%). The average age of male patients was 3.6 years old and the average age for female patients was 3.4 years old. The most common age of injury between the male and female genders was two years old (p<0.008) (Table 1).

**Table 1:** Demographics

	Count	N%
Age		
<1	71	3.3%
1	272	12.8%
2	522	24.6%
3	484	22.8%
4	244	11.5%
5	163	7.7%
6	140	6.6%
7	87	4.1%
8	72	3.4%
9	67	3.2%
Total	2122	100.0%
Sex		
Male	1269	59.8%
Female	853	40.2%
Total	2122	100.0%
Race		
Unknown	744	35.1%
White	938	44.2%
Black/African American	255	12.0%
American Indian/Alaska Native	4	0.2%
Native Hawaiian/Pacific Islander	2	0.1%
Asian	52	2.5%
Other	127	6.0%
Total	2122	100.0%

Ingestion was the most common diagnosis (p<0.01). Internal injuries were the most common body part injured (p<0.01). The most common locations of injury were home (38.6%), place of recreation or sport (20.3%), or other public property (7.3%). Dispositions included fatalities (2.7%), treated/examined and released (p<0.005, 49.0%), treated and transferred (3.3%), hospitalization (41.0%), held for observation (3.2%), and left AMA (0.8%) (Table 2).

**Table 2:** Disposition and Location ED: Emergency department; AMA: Against medical advice

	Count	N%
Disposition		
Treated and released, or examined without treatment	1039	49.0%
Treated and transferred to another hospital	70	3.3%
Treated and admitted for hospitalization	870	41.0%
Held for observation	68	3.2%
Left without being seen, Left AMA, Left without treatment, Eloped	18	0.8%
Fatality, includes being dead on arrival, died in the ED, and died after admission	57	2.7%
Total	2122	100.0%
Location		
Unknown or not recorded	714	33.6%
Home	819	38.6%
Farm/Ranch	0	0.0%
Street or highway	0	0.0%
Other public property	155	7.3%
Manufactured	0	0.0%
Industrial place	0	0.0%
School	4	0.2%
Place of recreation or sports	430	20.3%
Total	2122	100.0%

**Figure 1:**
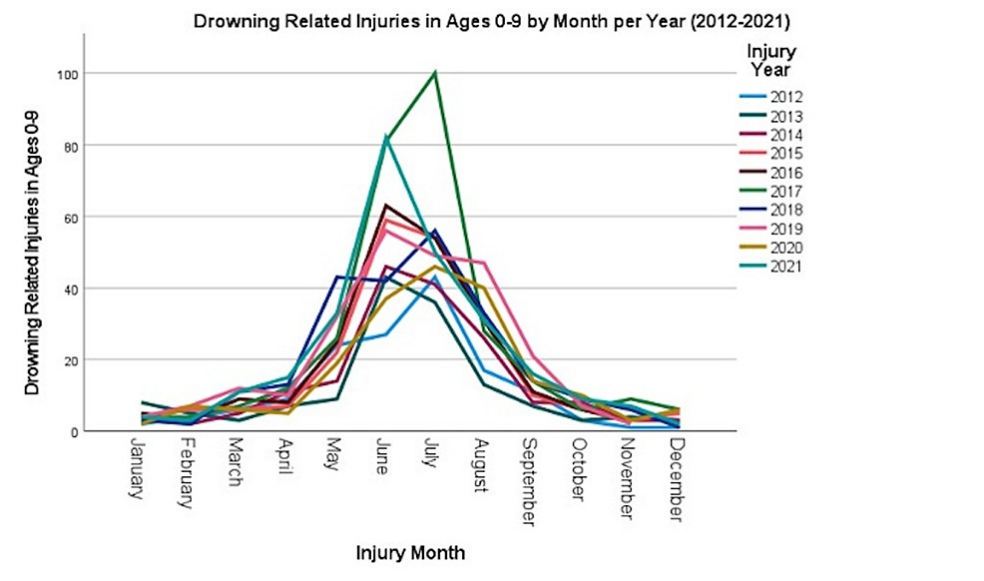
Injuries by Month Per Year

The months of May-August had the highest frequency of injuries reported, and 2021 had the highest frequency of injuries compared to all other years (Figure 1).

## Discussion

Results from this study reveal the disproportionate risk of drowning related incidents in young children, with those younger than the age of five years old representing 75% of the patients reported. Given our data highlights drowning related injuries between the years 2012-2021, it appears that despite injury prevention strategies made available by the AAFP, injuries remains high. This further underscores the importance of modifying current measures implemented to prevent such trauma related incidents that considers the added risk posed on this patient population in particular.

Our results on demographics not only echo nationally reported statistics by the Center for Disease Control and the literature in regards to age, but it also presents similar findings in terms of gender. Specifically, about 60% of the patients identified by this study were reported as males. Similarly, a study on the trends in US pediatric drownings between the years 1993 and 2008 conducted at the Center for Injury Research and Policy at Johns Hopkins Bloomberg School of Public Health reported similar rates of hospitalizations that were consistently greater for males compared to females [3]. As this trend has remained unchanged in more than 25 years of data samples, there is room for further extrapolation of the significance of this finding and ways to modify prevention strategies to help address this prevalence of drowning incidents in young males.

Aside from the emotional toll a trauma related incident can impose on those affected, including witnesses and loved ones, the long term neurological burdens and outcomes are life impacting, detrimental, and very understudied sequela of drowning related traumas. A 2013 study out of Helsinki conducted neurological and neuropsychological assessments on 21 patients that had drowned and been admitted to the pediatric ICU at a median age of 2.4 years. At the time of follow up, which occurred at median age 12.5, 57% of the children demonstrated neurological dysfunction and 40% had intellectual impairment represented by a full-scale intelligence quotient (FIQ) score of less than 80 [4]. Given the difficulty assessing these impacts early in the post hospitalization course and lack of long term follow up and management, the effect of these incidents could have broader implications not only on the child’s health outcomes but economic burdens and health care utilizations.

Trends in our study in terms of number of injuries per month revealed the highest incidence of drowning injuries took place between the months of May and August, an unsurprising finding given warmer months often set the stage for increased utilization of water related recreation. Loux et al. reported a similar finding in their retrospective study conducted at a large pediatric trauma center in Florida which showed that summer breaks and days with higher than average daily temperatures, both of which most often occurring between the months of May and August, as independently associated with a higher probability of drowning admissions [5]. Given this trend in seasonality, increased vigilance and preventative strategies tailored to higher risk times of the year are warranted.

Perhaps our most surprising finding was the sharp increase in drowning related injuries in the year 2021, marking one year post onset of the coronavirus disease 2019 (COVID-19) pandemic, a time when quarantine orders throughout the country kept children confined to their homes. Prior to this, the literature cited a decreasing trend in drowning incidents. A retrospective analysis of the 2003-2016 Health Care Cost and Utilization Project National Inpatient Sample and Kids’ Inpatient Database by Umapathi et al. demonstrated a decline of 31.5% in the annual incidence rate of drowning hospitalizations [6]. Similar findings were reported by Theodorou et al. from the University of California, Davis, where hospitalizations after drowning were assessed using the Kids Inpatient Database between 2000-2016. They found overall drowning hospitalizations decreased by 49% during this time period [7].

As many young children regularly visit their pediatrician for well child checks, it is a great time for pediatricians to evaluate drowning risks and educate parents on the necessary precautions. The Haddon Matrix Paradigm provides interventions related to personal, equipment, physical and social environment that can help reduce the risk of drowning and identify an accidental fall earlier [8]. One study also revealed that formal swimming lessons reduced the risk of drowning by 88% in children between the ages of 1-4, while informal swimming lesson did not show a significant reduction in drowning incidences [9]. This further highlights the importance of educating parents about enrolling their children in formal swimming lessons.

Additionally, in a qualitative study of Australian families of non-fatal drowning-related hospitalizations of children, the four key strategies recommended by families who had experienced non-fatal drowning included: supervision, restricting access to water through pool barriers, swimming lessons and cardiopulmonary resuscitation and response [10]. The American Academy of Pediatrics (AAP), highlights the importance of bystander cardiopulmonary resuscitation (CPR) as immediate resuscitation at the time of the submersion event prior to the arrival of emergency medical personnel as the most effective means to improve outcomes in the event of a drowning incident [11]. In addition, a retrospective study found that CPR performed by trained personnel resulted in better long-term outcomes for children in drowning accidents [12]. This thus emphasizes the importance of preventative measures and life-saving parental and supervisor education that can be provided during routine pediatric visits. Additionally, crucial conversations centering around adequate caregiver supervision can be held during pediatric visits. Although AAP recommends constant attentive supervision, Mackay et al. found that 19-22% of parents of one-four year olds in the USA left their child at a pool without supervision for more than two minutes [13]. Johnson et al. found that 38% of parents reported that they would leave their toddler in the pool to check their phone outside of the pool, and 39% of parents partaking in the study reported that they would run inside to take a bathroom break while their toddler is in the pool [14]. Even the importance of the AAP recommendation of arm’s reach supervision was recently found to be regarded as varying in importance to parents [15]. These critical conversations surrounding specific recommendations should be shared with parents with an emphasis on their effectiveness and ability to avert fatal and otherwise preventable accidents to their own children. 

Counseling is also more pertinent in households with an ungated/accessible pool. However, it is also important to note that socio demographic factors may influence the ability to access swimming lessons. Limited funding for community swimming pools and swimming programs restricts access to many impoverished communities. Resources for low-cost or free lessons can help reduce the financial barrier that keeps high risk children from learning the necessary skills [11]. Although the primary focus has been on preventative education surrounding drownings in a pool setting, it is important to note that for toddlers and infants the majority of these unfortunate accidents were in locations such as bathtubs, demonstrating the importance of preventative drowning education regardless of pool accessibility.

## Conclusions

All in all, resources and preventative strategies exist to combat drowning; however, a joint effort between pediatricians and families must be explored in order to ensure patients and their families are receiving the proper education and having the necessary conversations to prevent drowning incidents. This is especially true now as we move towards a post-COVID-19 era where things like remote learning and work-from-home economies keep kids at home for longer periods of time. Given the discussions posed by previous studies on drowning and our study’s finding of an isolated increase in drowning-related injuries in the post-COVID-19 year of 2021, further investigation into how we can readdress prevention strategies has become essential.
